# AAV-Mediated Cone Rescue in a Naturally Occurring Mouse Model of CNGA3-Achromatopsia

**DOI:** 10.1371/journal.pone.0035250

**Published:** 2012-04-11

**Authors:** Ji-jing Pang, Wen-Tao Deng, Xufeng Dai, Bo Lei, Drew Everhart, Yumiko Umino, Jie Li, Keqing Zhang, Song Mao, Sanford L. Boye, Li Liu, Vince A. Chiodo, Xuan Liu, Wei Shi, Ye Tao, Bo Chang, William W. Hauswirth

**Affiliations:** 1 Department of Ophthalmology, College of Medicine, University of Florida, Gainesville, Florida, United States of America; 2 Eye Hospital, School of Optometry and Ophthalmology, Wenzhou Medical College, Wenzhou, China; 3 Chongqing Key Laboratory of Ophthalmology, Ophthalmology, The First Affiliated Hospital of Chongqing Medical University, Chongqing, China; 4 Ophthalmology, SUNY Upstate Medical University, Syracuse, New York, United States of America; 5 The Jackson Laboratory, Bar Harbor, Maine, United States of America; University of Rochester, United States of America

## Abstract

Achromatopsia is a rare autosomal recessive disorder which shows color blindness, severely impaired visual acuity, and extreme sensitivity to bright light. Mutations in the alpha subunits of the cone cyclic nucleotide-gated channels (*CNGA3*) are responsible for about 1/4 of achromatopsia in the U.S. and Europe. Here, we test whether gene replacement therapy using an AAV5 vector could restore cone-mediated function and arrest cone degeneration in the *cpfl5* mouse, a naturally occurring mouse model of achromatopsia with a *CNGA3* mutation. We show that gene therapy leads to significant rescue of cone-mediated ERGs, normal visual acuities and contrast sensitivities. Normal expression and outer segment localization of both M- and S-opsins were maintained in treated retinas. The therapeutic effect of treatment lasted for at least 5 months post-injection. This study is the first demonstration of substantial, relatively long-term restoration of cone-mediated light responsiveness and visual behavior in a naturally occurring mouse model of CNGA3 achromatopsia. The results provide the foundation for development of an AAV5-based gene therapy trial for human *CNGA3* achromatopsia.

## Introduction

The human retina contains about 6 million cone photoreceptors, which are responsible for fine resolution, central and color vision. The distribution of cones increases from peripheral retina to central macula or fovea that is comprised nearly 100% of the cones. Achromatopsia is a relatively rare, autosomal recessive congenital retinal disorder that is characterized by cone dysfunction. There are two clinical forms of achromatopsia: incomplete and complete. Patients with incomplete achromatopsia display a milder phenotype and retain residual cone function. Individuals with complete achromatopsia suffer from severely reduced visual acuity, pendular nystagmus, photophobia and color blindness. Since the only functional photoreceptors in complete achromatopsia are rods, which are more sensitive to light, or become saturate at higher levels of illumination, these individuals experience extreme light sensitivity and daylight blindness [1]–[3].

In humans, four genes that all encode essential components in the cone phototransduction cascade have been identified to cause achromatopsia. Mutations in the two subunits (CNGA3, CNGB3) of cone photoreceptor cyclic nucleotide-gated (CNG) channels account for approximately 75% of all cases of complete achromatopsia [1], [4]–[9]. Some of the remaining cases are caused by mutations in alpha subunit of cone transducin (GNAT2) [10], [11] and the catalytic alpha subunit of cone phosphodiesterase (PDE6C) [12], [13]. In cone photoreceptors, CNG ion channels are integral tetrameric plasma membrane proteins composed of two A3 and two B3 subunits. Photoreceptors respond to light by closure of the CNG channels induced by hydrolysis of cGMP, resulting in membrane hyperpolarization and decreased synaptic glutamate release [1], [14]. A3 and B3 are related proteins composed of six transmembrane helices (S1–S6), a pore-forming region between S5 and S6, a cyclic nucleotide-binding domain (CNBD), and a C-linker region between S6 and CNBD, which mediates channel gating [15], [16].

The majority of mutations in the *CNGA3* gene in human patients identified so far are missense mutations, suggesting that CNG channel function has little tolerance for amino acid substitutions [6], [9], [17]. Missense mutations that ablate CNG channel function typically occur at amino acid residues conserved among the members of the CNG channel family. The majority of these changes are located within structurally and functionally important regions of the CNGA3 polypeptide, i.e., the transmembrane helices, the pore, and the cGMP-binding domain [9].

A new cone photoreceptor function loss 5 (*cpfl5*) mouse strain recently discovered at The Jackson Laboratory exhibits an ocular phenotype similar to complete human achromatopsia. The *cpfl5* mouse carries a missense mutation in exon 5 of the *Cnga3* gene [18]. A single nucleotide A to G change at position 492 in exon 5 results in a substitution of alanine for threonine. The *cpfl5* mouse exhibits selective loss of cone-mediated light responses accompanied by cone cell loss, similar to the phenotype of complete achromatopsia patients with *Cnga3* mutations. Our early study with an AAV5 vector containing a human blue cone promoter (HB570) showed partial restoration of cone function up to 2 months in cpfl5 mouse [18]. Others have shown partial restoration of cone function in a CNGA3^-/-^ knock out mouse model using a capsid mutant AAV5 vector containing a mouse S-opsin promoter [19]. However, in both studies, cone function and cell survival were evaluated in a relatively short-term [18], [19]. Moreover, in the later study, photopic b-wave responses were restored only to an average of 1/3 of normal amplitudes at 10 weeks post-injection. Here we report 5-month preservation of cone structure and function in the cpfl5 mouse, a natural missense *Cnga3* mutant, when the therapeutic transgene is driven by a CBA promoter in an AAV5 vector.

## Results

### AAV-mediated CNGA3 Expression in cpfl5 Mice

**Figure 1:**
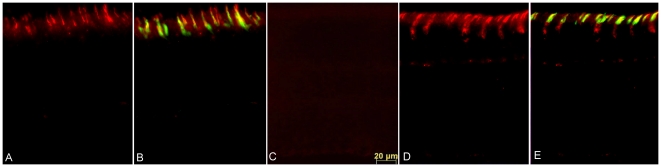
AAV5-CBA-m*Cnga3* leads to CNGA3 expression specifically in cone photoreceptors. A. CNGA3 expression (red) in a normal C57BL/6J mouse. B. Same field as (A) with PNA staining (green) to show CNGA3 expression in cones. C. No CNGA3 expression was found in an untreated *cpfl5* eye. D. AAV-mediated expression of CNGA3 (red) in cone photoreceptors of the partner treated *cpfl5* eye. E. Same field as (D) with PNA staining (green) to show CNGA3 expression in cones. OS, outer segments; IS, inner segments.

Gene therapy in the *cpfl5* mouse was tested by subretinal delivery of the mouse *Cnga3* gene driven by the ubiquitous CBA promoter packaged into an AAV5 vector. Subretinal injection was performed at P14 before significant cone photoreceptor degeneration had initiated. As seen in normal mice (Fig. 1A, 1B), immunostaining showed robust CNGA3 expression in the cone outer segment (OS) layer of treated *cpfl5* retina (Fig. 1D) 5 months following treatment with AAV5-CBA-m*Cnga3* whereas the contralateral uninjected eye lacked detectible CNGA3 labeling (Fig. 1C). Colocalization of CNGA3 and cone-specific lectin peanut agglutinin (PNA) staining suggests that CNGA3 expression is targeted primarily to cone outer segments (Fig. 1E), and a small amount was detected in cone cell bodies.

### Cone Opsin Expression and Localization were Restored in *cpfl5* Mice

**Figure 2:**
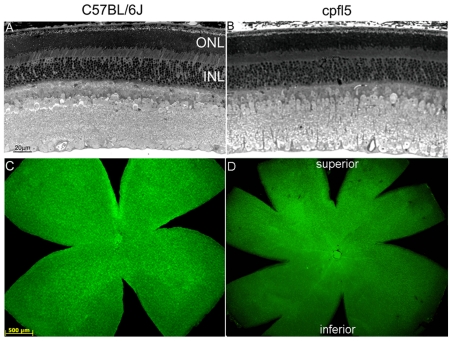
Cone photoreceptors degenerate in *cpfl5* mice. Representative light microscopic images of retinal paraffin sections from the superior hemisphere of a normal 5 month old C57BL/6J (A) and an age matched *cpfl5* mouse (B) showing similar appearing outer and inner nuclear layers. Whole mount PNA staining (green dots) of a 5 month C57BL/6J retina (C, right eye): and a *cpfl5* retina at 5 months (D, left eye) showing that cones outside the superior hemisphere, primarily in the nasal inferior quadrant of the *cpfl5* retina where most of the S-cones are located have degenerated. Panels A and B and Panels C and D are at the same scale.

**Figure 3:**
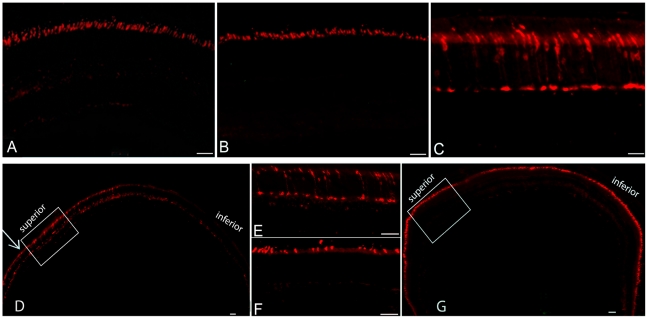
AAV-mediated CNGA3 expression restores expression and localization of M-opsin in *cpfl5* mice. By immunostaining, a 3-week-old C57 BL/6J wild type control retinal section (A) and an age-matched *cpfl5* retina (B) has identical expression and localization of M-opsin in the outer segments. In contrast, a section of a 10-week-old *cpfl5* mouse (C) shows that M-opsin has become mislocalized to cone inner segments, nuclei, and synaptic termini. Upon CNGA3 vector treatment, M-opsin expression and localization is nearly normal (D-G). Retinal sections from untreated (D and E, E is inset of D) and the contralateral treated (F, G, F is an insert of G) *cpfl5* eyes at 5 months post-treatment show that M-opsin can be detected only in the superior region (arrow) of the retina in an untreated 5-month-old *cpfl5* mouse (D); in addition, the remaining M-opsin in superior (dorsal) region is mislocalized (E). In contrast, intense M-opsin staining was detected throughout the entire retina in the contraleteral vector-treated eye (G) and was exclusively localized to the cone outer segments (F). Scale bars are equal to 50 µm.

**Figure 4:**
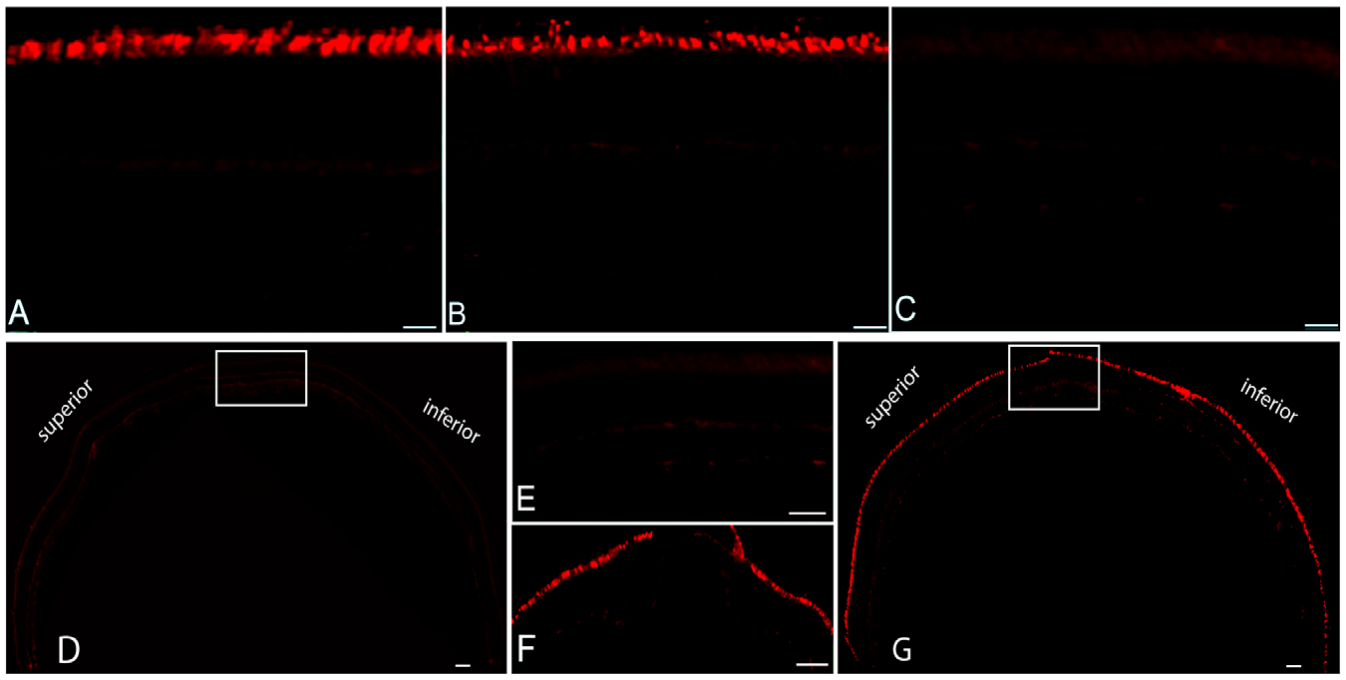
AAV-mediated CNGA3 expression maintains expression and localization of S-opsin in **cpfl5** mice. Retinal sections immunostained with S-opsin antibody from 3-week-old C57 BL/6J wild type control mouse (A) and an age-matched *cpfl5* retina (B) showing robust S-opsin expression in cone outer segments. Section from a 10-week-old *cpfl5* retina (C) showed no S-opsin staining indicating that profound S-cone degeneration had occurred. Five months after vectored CNGA3 expression, a treated *cpfl5* retina maintained normal expression and localization of S-opsin immunostaining (G and inset F) for at least 5 months in contrast to the contralateral untreated retina (D and E). Scale bars equal 50 µm.

It has been shown previously that loss of CNGA3 resulted in impaired expression and trafficking of cone opsins as well as cone cell death in the *Cnga3* ^-/-^ mouse [20]. Here we documented the retinal morphology and expression patterns of M- and S-cone opsins in *cpfl5* mice (Fig. 2). 5-month-old *cpfl5* mice (Fig. 2B) exhibited photoreceptor outer & inner segment lengths and outer nuclear layer thickness similar to normal C57 BL/6J mice (Fig. 2A). However, retinal wholemounts stained with cone outer segment sheath specific PNA (green) revealed that moderate cone structure loss had occurred at this time point, primarily in the inferior (ventral) and nasal regions of the retina (Fig. 2D). Cone densities in these regions (Fig. 2D) have decreased compared to the dorsal (superior) and temporal regions of the normal retina (Fig. 2C). Expression and localization of cone opsins were then analyzed at different ages in *cpfl5* eyes by immunostaining retinal sections with antibodies to M- or S-opsin. 3-week-old *cpfl5* mice (Fig. 3B) exhibited M-opsin staining intensity and localization to OS consistent with that of wild type controls (Fig. 3A). However, by 10 weeks of age, M-opsin was mislocalized to the inner segment, cone nuclei, and cone pedicles (Fig. 3C). The loss of S-opsin staining proceeded more rapidly than M-opsin. In 3-week-old *cpfl5* mice (Fig. 4B), the intensity of S-opsin immunostaining started to decrease (Fig. 4A), but still localized in the OS. However, by 10 weeks of age, no S-opsin staining was seen in *cpfl5* mice indicating that profound S-cone OS loss had occurred (Fig. 4C).

To evaluate whether AAV5-CBA-m*Cnga3* treatment could arrest cone opsin loss and maintain its normal OS localization following P14 treatment, we analyzed retinal sections from untreated and contralateral treated *cpfl5* eyes with M- and S-opsin antibodies at 5 months after treatment. In untreated eyes, M-opsin can be detected in the superior retinal hemisphere but rarely in the inferior hemisphere (Fig. 3D). This is consistent with PNA staining showing that cones are preserved in the superior region at this age (Fig. 2B). As noted above however, the remaining M-opsin was mislocalized to the inner segments, cone nuclei, cone pedicles (Fig. 3E). In contrast, in vector-treated contralateral eyes, more intense M-opsin staining was detected throughout the retina (Fig. 3G), with an exclusive OS localization pattern (Fig. 3F). In untreated eyes, virtually no S-opsin staining can be detected in any part of the retina (Fig. 4D, 4E) whereas in treated eyes S-opsin was detected throughout the retina and was localized primarily in the cone OS (Fig. 4G, 4F). In summary, AAV5-CBA-Cnga3 P14 treatment prevented cone degeneration and maintained normal M- and S-opsin expression and localization in the *cpfl5* mice for at least 5 months.

### Cone Function is Restored in AAV-treated *cpfl5* Mice

**Figure 5:**
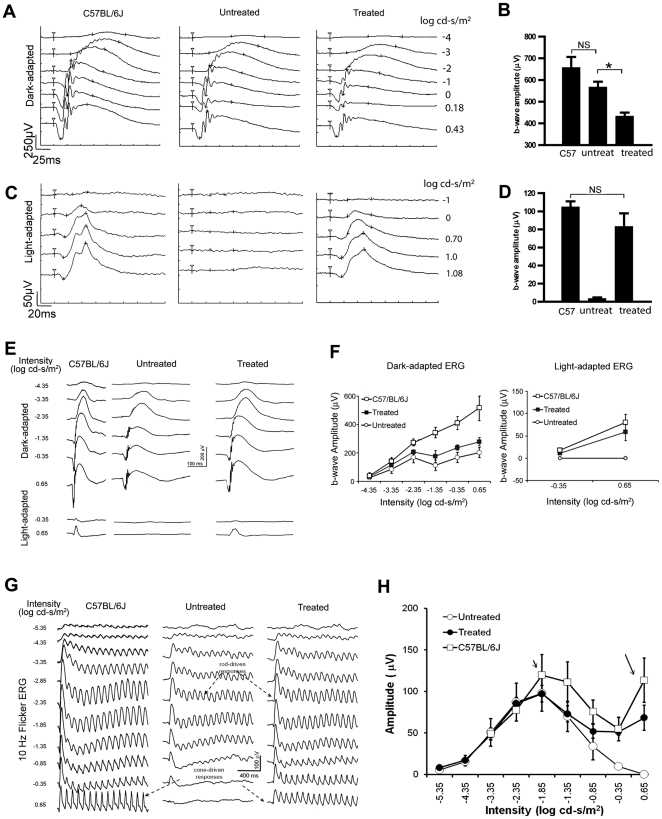
AAV5-CBA-m*Cnga3* treatment restores cone-mediated ERG. A-D. Single-stimulus ERG recordings from *cpfl5* mice 3 weeks post-treatment compared with normal C57BL/6J controls. Representative rod-mediated (A) ERG responses and statistical analysis (B) of b-wave amplitudes at 0.43 log cd-s per m^2^. Representative cone-mediated ERG (C) responses and statistical analysis (D) of b-wave amplitudes at 1.08 log cd-s per m^2^. n = 3 for each group. G-H. 10 Hz flicker ERG recordings from *cpfl5* mice 5 months post-treatment compared with normal C57BL/6J controls. Representative dark-adapted 10Hz flicker ERG (G) from normal C57 BL/6J, untreated and the contralateral treated *cpfl5* eyes. Statistical analysis (H) showed that the cone-mediated ERG amplitudes were restored up to 60% of wild type levels for at least 5 months post-treatment; short arrow: pure rod-mediated responses; long arrow: pure cone-mediated responses. NS: No statistical difference; *: P<0.05.

We find that *cpfl5* mice treated with AAV5-CBA-m*Cnga3* vector significantly restored cone photoreceptor function as measured by both single-stimulus ERG and 10 Hz flicker ERG recordings. Dark- and light-adapted single-stimulus ERG analyses were initiated at 3 weeks post-treatment (Fig. 5A, 5B), and repeated regularly until 5 months post-treatment. At 3 weeks post-injection, the average light-adapted b-wave amplitude (Fig. 5C, 5D) was 82 ± 16 µV (n = 3) in treated eyes, about 80% of the wild-type controls (652 ± 55 µV, n = 3, P = 0.09) at a stimulus intensity of 1.08 log cd-s/m^2^. No ERG responses were detected in untreated eyes (2 ± 2.6 µV, n = 3). Although the untreated cpfl5 (561 ± 31 µV) and normal C57BL/67 eyes (652 ± 55 µV) had statistically similar dark-adapted b-wave amplitudes (n = 3, P = 0.19), there was a reduction in the average of dark-adapted b-wave amplitude in treated eyes (427 ± 23 µV at 0.43 log cd-s/m^2^) vs. in untreated eyes (561 ± 31 µV, n = 3, P< 0.05) (Fig. 5A, 5B. This decreased rod-mediated ERG amplitude upon treatment is likely the consequence of injection-related damage since this difference diminished (P>0.05) when the same mice were similarly analyzed at 5 months post-injection (Fig. 5F, 5H). We have consistently observed that rod-mediated ERG responses in 5.5 months old untreated cpfl5 eyes were lower than the age-matched wild-type controls (P<0.05), and the reason is under investigation.

At 5 months post-injection, the average light-adapted ERG response in treated cpfl5 was 58.40 ± 18.87 µV (n = 3), about 70% of the wild-type controls at a stimulus intensity of 0.65 log cd-s per m^2^ (Fig. 5E, 5F). 10 Hz flicker ERG (Fig. 5G, 5H) showed that untreated and treated *cpfl5* eyes at lower stimulus intensities (≤ -1.85 log cd-s per m^2^) which reflect pure rod-mediated responses [21], were similar. At high stimulus intensities (> -1.85 log cd-s per m^2^), cone-driven responses in treated eyes showed much higher amplitudes than partner untreated eyes. The average b-wave amplitudes plotted against light intensities from untreated, contralateral treated, and wild type control eyes (Fig. 5H) showed a cone peak of 68.29 ± 15.19 µV in the treated eyes at 0.65 log cd-s/m^2^ whereas a signal is undetectable in untreated eyes (n = 3, P < 0.01). These cone ERG responses in the treated eyes were about 60% of that recorded in the same session from uninjected wild type controls (113.68 ± 26.61 µV, n = 5). In summary, cone-mediated ERG responses were restored and maintained for at least 5 months in AAV5-CBA-m*Cnga3* treated eyes.

### Restoration of Cone-mediated Acuity and Contrast Sensitivity in *cpfl5* mice

**Figure 6:**
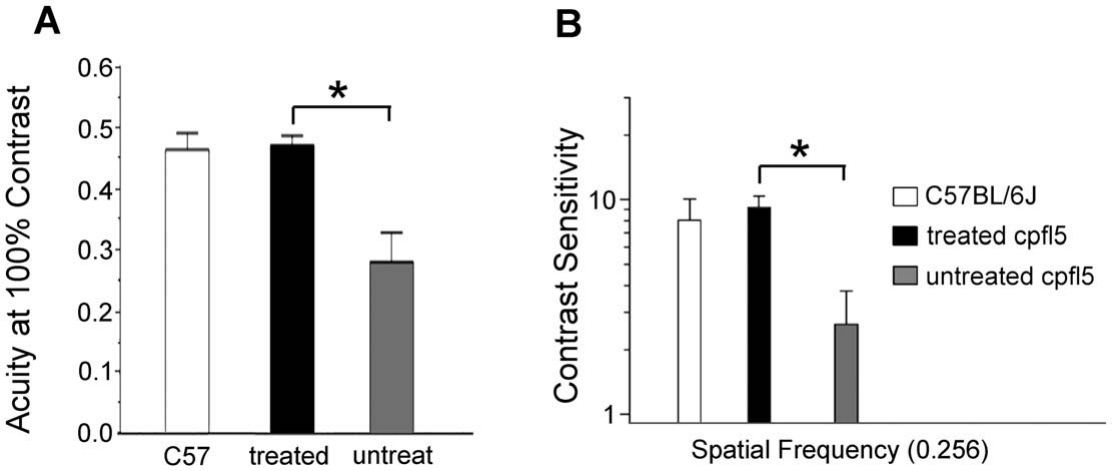
Rescue of visual acuity and contrast sensitivity in *cpfl5* mice. Comparison of average values for photopic acuities (A) and contrast sensitivities (B) from untreated, treated, and wild type mice (n = 4 for each group) 5 months post injection. Values are expressed as the mean ± SD for each group (* P < 0.05 for acuity and P<0.01 for contrast sensitivity).

We also tested whether maintenance of cone structure, normal cone opsin localization and restoration of cone ERG function translated into improvement in cone-mediated visual behavior by measuring optomotor responses to rotating sine-wave gratings [22], [23]. Optomotor behavioral analysis revealed significant improvement in cone-mediated acuity and contrast sensitivity of AAV-treated *cpfl5* eyes. Untreated eyes displayed poor visual acuities of 0.273 ± 0.111 cycles per degree whereas in treated eyes, acuities were 0.457 ± 0.059 cycles per degree (Fig. 6A), a level essentially identical to that in wild type controls (0.477 ± 0.026) and significantly better than untreated cpfl5 eyes (n = 4, P<0.05). In parallel, contrast sensitivities tested at a spatial frequency of 0.256 cycles/degree in treated eyes (9.20 ± 2.30, n = 4) showed similar contrast thresholds as wild type controls (8.49 ± 2.78, n = 4), and were significantly better than untreated eyes (2.34 ± 0.74, P<0.01, n = 4; Fig. 6B). Thus, cone-mediated visual acuity and contrast sensitivity were restored to wild type levels, demonstrating a positive therapeutic effect on visual behavior in treated *cpfl5* mice.

## Discussion

We demonstrate here that AAV5-CBA-mCnga3-mediated gene replacement therapy restored cone-specific ERG, cone-mediated visual acuity and visual contrast in the *cpfl5* mouse, a naturally occurring mouse model of achromatopsia caused by *Cnga3* deficiency. We also showed that gene therapy restored normal levels and localization of cone opsins. These therapeutic effects were maintained for at least 5 months after treatment.

We previously reported that AAV5-mediated gene therapy using the HB570 human blue cone opsin promoter (HB570) [18], [24] or human red/green opsin promoter (PR2.1) (our unpublished work) leads to partial restoration of both S- and M- cone visual function in cpfl5 mice. A recent study using an AAV5 vector with a surface exposed tyrosine to phenylalanine mutation on the capsid (Y719F) containing a mouse S-opsin promoter also demonstrated partial cone functional restoration in a CNGA3^-/-^ mouse model 11 weeks after injection [19]. In that study photopic b wave responses were on average 1/3 the amplitude of matched wild type controls. Compared to both previous studies, our current results using the strong, fast acting CBA promoter showed more robust and longer term restoration of ERG amplitudes, and vision elicited behavior.

Based on immunostaing using a CNGA3-specific antibody, CNGA3 expression driven by CBA promoter was primarily restricted to cone photoreceptors of cpfl5 retina with majority of CNGA3 found in the cone OS where it is normally localized, suggesting that off-target expression is inefficient, likely due to a posttranscriptional process mediating the instability of CNGA3 protein in non-cone cells. This phenomenon is not unexpected, particularly when the treatment is longer than 2–3 months. Similar observations of cell specific expression have also been found when using CBA or a truncated version of CBA (smCBA) to express RPE65 in the RPE cells [25],GC1 [26] and PDEβ proteins [27], [28] in photoreceptors. In addition, the *RPE65*-LCA clinical trials have demonstrated the ability of the CBA promoter to support therapeutic transgene expression in the retina and restoration of visual function [29]–[33]. Although a cone-specific promoter might be preferred if off-target expression from a CBA promoter is a concern, it remains unclear which promoter would be best for transducing all cone subtypes. The human blue cone promoter (HB570) [18] or 2.1 kb human red/green opsin promoter (PR2.1. unpublished work) used in our previous cpfl5 mice studies, or the mouse S-opsin promoter used in the CNGA3 knockout mice study [19] have not been investigated in non-human primates. In fact, when evaluated in dog, the HB570 promoter did not drive expression in S-cones, only in M/L cones. Therefore, the current study using the CBA promoter combined with AAV5 serotype might remain the preferred combination of promoter and AAV serotype.

GFP expression in photoreceptor cells from AAV5-CBA-GFP vector can be detected as early as 7 days after subretinal injection [34]. Early onset AAV5 expression is also supported by the fact that almost half the magnitude of the final cone ERG restoration is obtained one week after P14 injection with AAV5-CBA-*Cnga3* vector (data not shown). It takes about 3 weeks for the AAV5-CBA- *Cnga3* vector to express sufficient CNGA3 protein to restore a stable cone ERG function (Figure 1A &1B). Cone opsins are almost normal in untreated 3-week-old cpfl5 mice (Figure 3 & 4) and cone structure is intact by whole mount PNA staining at P35 (data not shown). That explains why we can maintain most of the cone opsins 5-month after P14 treatment.

The rapid loss of S-opsin labeling in cpfl5 mouse suggests treatment earlier than P14 might lead to even more recovery of cone function, however, it is difficult to detach a significant fraction of the mouse retina prior to eye opening at P14 via trans-cornea subretinal injection without substantial injection-related damage [27], [34]. We have noted a close correlation between maximal stable rescue and the extent of retinal coverage by the vector. For example, P14 treatment [27] was more therapeutically robust than P2 treatment in the rapid degenerating rd10 mouse that carries a PDEβ mutation [35]. Unlike the human retina, in which of the density of cones is very high in the central macula (fovea), M- and S-cones in the mouse retina are relatively evenly distributed in fixed topographic patterns across the entire retina. Currently, the most reliable way to evaluate successful cone therapy in mouse would be to limit analysis to eyes where the majority of cone photoreceptors were transduced, i.e. in retinas in which subretinal vector had detached all or nearly all of the retina. We found one microliter of vector sufficient to detach nearly the entire mouse retina, resulting in a homogenous “shallow bleb”, which appears to minimize subsequent retinal detachment-related photoreceptor loss. From our previous work on cone therapy in the rd12 (mutant RPE65) mouse, we found that a single subretinal injection of AAV-RPE65 can restore cone ERG amplitudes to about 2/3 of wild type levels [25], similar to that reported here. This approach may therefore, in part, explain the better cone ERG rescue reported here than previously [19], in which subretinal vector detached only 30% of the retina.

It has been shown that AAV vectors with capsid surface exposed tyrosine residues mutated to phenylalanine (Y-F) have increased transgene expression levels and transduction kinetics relative to the corresponding standard wild-type AAV vectors [36]. An AAV8 Y-F capsid mutant vector demonstrated more effective and longer therapeutic effect compared to standard AAV8 and AAV5 in the rd10 mouse model of retinal degeneration [28]. A side by side comparison of AAV5 and the AAV5 (Y719F) utilized in the Michalakis et al. study [19] was not presented, therefore, it is uncertain whether there is any advantage of AAV5 (Y719F) over unmutated AAV5 for achieving cone rescue. In our case, perhaps the robust nature of CBA expression precluded the need for an AAV5 capsid variant. It may also be that the differences in level of rescue are related to the fact that the two studies were performed in different mouse models of CNGA3 deficiency.

Both cpfl5 and CNGA3^-/-^ mice display the essential hallmarks of achromatopsia observed in human patients, in which cone responses are absent whereas rod function is retained [18], [20], [37]. In both mouse strains, cones gradually degenerated, possibly as a result of the impaired phototranduction cascade or the structural instability of cone outer segments [20]. Both models displayed defective cone opsin transport and failure to target cone opsin to the outer segments. Cones in the dorsal (superior) hemisphere containing mainly M-opsin survived significantly longer than ventral (inferior) cones with primarily S-opsin. Interestingly, the same pattern of S-cone loss preceding M-cone loss has been observed in other mouse models of retinal degeneration [38], [39].

In 5-month-old cpfl5 eyes, M-opsin can be only detected in superior region of the retina, and is mislocalized to inner segments and cone cell bodies (Fig. 3D). However, M-cone structure in superior retina is still relatively intact as shown by cone outer segment sheath specific PNA staining (Fig. 2D).It would be interesting to test whether delayed treatment in older cpfl5 mice would still lead to rescue of the remaining M-cones as has been seen with delayed treatment of rd12 mice [38]. If so, correct intracellular M-opsin distribution should also be restored since cone cell bodies are still be present although cone functional degeneration has occurred early. This would have important implications for human *Cnga3* achromatopsia which is characterized by a gradual and variable degree of cone loss [9], [40].

In summary, we demonstrate restoration of cone function for at least 5 months in a naturally occurring mouse model of *Cnga3* achromatopsia using an AAV5 vector. Both M-cone and S-cone degeneration was prevented and correct trafficking of cones opsins restored. As mutations in CNG channels are the most common cause of human achromatopsia (CNGB3 ≥ 50%, CNGA3 ≥ 25%), the demonstration of successful gene therapies by AAV vectors to rescue cone function in this model [18], [41], in CNGB3-mutant dogs [42] and in *Cnga3* ^-/-^ mice [19] provides key proof-of-principle for future achromatopsia clinical trials in humans.

## Materials and Methods

### Animals

*Cpfl5* mice and the isogenic wild type strain C57BL/6J mice were obtained from The Jackson Laboratory (Bar Harbor, ME). All mice were bred and maintained in the University of Florida Health Science Center Animal Care Services Facilities under a 12hr/12hr light/dark cycle. All experiment protocols were approved by the University of Florida Institutional Animal Care and Use Committee and conducted in accordance with the Association for Research in Vision and Ophthalmology (ARVO) Statement for the Use of Animals in Ophthalmic and Vision Research and National Institute of Health (NIH) regulations.

### Construction of the AAV5-CBA-m*Cnga3* Vector

Serotype 5 AAV vectors were used in this study as they have been demonstrated previously to mediate robust transduction efficiency and relatively rapid expression onset in photoreceptors [43]. A Mammalian Gene Collection (MGC) clone that contains full length mouse *Cnga3* cDNA was purchased from Invitrogen (Carlsbad, CA). NotI restriction enzyme sites were added to both ends of the cDNA by PCR utilizing forward 5′ –TTAGCGGCCGCGCAGAGATGGCAAAGGTGA- 3′ and reverse 5′ –TTAGCGGCCGCTGCATTTTCAGTCAGTCTTTGAA-3′ primers. The PCR fragment was then cloned into pCRblunt plasmid (Invitrogen) and sequence verified. Using the AAV vector plasmid pTR-CBA-hGFP, containing the CBA promoter driving expression of GFP, the *hGFP* cDNA was replaced with *mCnga3* via NotI digestion. AAV vectors were packaged and tittered according to previously published methods [44], [45].

### Subretinal Vector Injection

One microliter of AAV5-CBA-m*Cnga3* vector containing 10^10^ total DNAse resistant vector genomes was injected subretinally into the left eye of each P14 *cpfl5* mouse (n = 30) and the right eyes remained uninjected as controls. Subretinal injections were performed as described previously [27], [28], [38], [39]. Only those mice that had more than 95% retinal detachment and minimal complications following subretinal injections were kept for further evaluation [25]. Eighteen cpfl5 mice (60% of total) met this criterion, which resulted in at least three animals for each experiment.

### Electroretinographic Analysis

Initial ERG responses of *cpfl5* mice and isogenic wild type controls were recorded at 3 weeks following injection, and repeated at the 3^rd^ and 5^th^ month post-treatment using a Toennies Multiliner Vision instrument (Höchberg, Germany) according to protocols described previously [26], [39], [46]. Briefly, mice were dark adapted overnight and anesthetized with a mixture of 100 mg/kg ketamine, 20 mg/kg xylazine and saline at a ratio of 1:1:5. Full field ERGs were recorded using gold wire loop electrodes placed on each cornea and a reference electrode placed subcutaneously between the eyes. Scotopic rod recordings were performed with seven increasing light intensities of white light between 0.01 mcds/m^2^ and 5 cds/m^2^. Ten responses were recorded and averaged at each light intensity. Photopic cone recordings were done after mice were light adapted to a white background light of 100 cds/m^2^ for 5 min. Recordings were performed with five increasing flash intensities between 100 mcds/m^2^ and 12 cds/m^2^ in the presence of a constant 100 mcds/m^2^ rod suppressing background light. Fifty responses were recorded and averaged at each intensity. Photopic b-wave amplitudes from untreated, treated *cpfl5* and wild type eyes at each intensity were averaged and compared by repeated-measures ANOVA with the Bonferroni post hoc test for ANOVA (*P* < 0.05) to compare means at individual flash intensities.

For flicker ERG responses at 5 months after P14 treatment, untreated, treated *cpfl5* and wild type control eyes were analyzed with a custom-designed ERG system with a Ganzfeld illumination using Grass PS22 Xenon visual stimulator (Grass Instrument Inc. West Warwick, RI). Procedures were performed as described previously [39]. ERG data are presented as mean ± standard deviation (mean ± SD). Statistical significance was examined with ANOVA as above. Pairwise comparisons between groups for the ERG were performed by the Bonferroni post hoc test (*P < 0.05)*.

### Optomotor Testing

Photopic and scotopic visual acuities and contrast sensitivities of treated and untreated mouse eyes were measured using a two-alternative forced choice paradigm as described previously [22], [23], [26], [39] with minor modifications. Acuity was defined as the highest spatial frequency (100% contrast) yielding a threshold response, and contrast sensitivity was defined as 100 divided by the lowest percent contrast yielding a threshold response (sinusoidal pattern at 0.256 cycles/degree). For both photopic and scotopic acuity, the initial stimulus was a 0.200 cycles/degree sinusoidal pattern with a fixed 100% contrast. For photopic contrast sensitivity measurements, the initial pattern was presented at 100% contrast, with a fixed spatial frequency of 0.256 cycles/degree. For scotopic contrast sensitivity measurements, the spatial frequency was fixed at 0.031 cycles/degree, a spatial frequency tuned for rod vision [22]. All patterns were presented at a speed of 12 degrees per second. Photopic vision was measured at a mean luminance of 70 cd/m^2^. For scotopic measurements, mice were dark-adapted overnight and light levels were attenuated to 3.5 × 10–^5^ cd/m^2^ through the use of neutral density filters. Visual acuities and contrast sensitivities were measured for both eyes of each mouse four to six times over a period of 1–2 weeks. Wild type control animals were 6 months old at testing time (n = 5), and P14 treated animals were at least 5.5 months old (n = 5). Unpaired t-tests were carried out on acuity and percent contrast values to determine significance of results.

### Tissue Preparation and Immunohistochemistry

Eyes were enucleated at 5 months after P14 treatment. Retinal sections were prepared according to previously described methods [34], [39]. Briefly, immediately following sacrifice, the limbus of injected and uninjected eyes was marked at “12 o’clock” with a hot needle which facilitated orientation. The eyes were then enucleated and fixed in 4% paraformaldehyde overnight. The cornea and lens were then removed from each eye without disturbing the retina. The remaining eyecup was rinsed with PBS and then cryoprotected by placing it in 30% sucrose in PBS for 4 hours at 4°. Eyecups were then embedded in cryostat compound (Tissue TEK OCT, Sakura Finetek USA, Inc., Torrance, CA) and frozen at - 80°C.

Retinal cryosections were cut at 12µM thickness, then rinsed in PBS and blocked in 2% normal goat serum, 0.3% Triton X-100 in 1% BSA in PBS for 1 hour at room temperature. Lectin PNA conjugated to a Alexa Fluor 488 (1: 200, L21409, Invitrogen), S-cone opsin, M-cone opsin primary antibodies (1:200, Santa Cruz Biotechnology, Santa Cruz, CA), or rabbit polyclonal CNGA3 (generously provided by Dr. Martin Biel (Ludwig-Maximilians-Universität, München) was diluted to 1:400 in 0.1% Triton X-100 and 1% BSA in PBS, and incubated overnight at 4°C. The sections were then washed 3 times with PBS, then incubated with IgG secondary antibody tagged with Alexa-594 (molecular Probes, Eugene OR) diluted 1:500 in PBS at room temperature for 1 hour and washed with PBS. Sections were mounted with Vectashiled Mounting Medium for Fluorescence (H-1000, Vector lab, In. Burlingame, CA) and coverslipped. Sections were analyzed with a Zeiss CD25 microscope fitted with Axiovision Rel. 4.6 software. All fluorescent images were acquired using identical exposure settings.

## References

[1] Kohl S, Varsanyi B, Antunes GA, Baumann B, Hoyng CB (2005). CNGB3 mutations account for 50% of all cases with autosomal recessive achromatopsia.. Eur J Hum Genet.

[2] Michaelides M, Hunt DM, Moore AT (2004). The cone dysfunction syndromes.. Br J Ophthalmol.

[3] Michaelides M, Hardcastle AJ, Hunt DM, Moore AT (2006). Progressive cone and cone-rod dystrophies: phenotypes and underlying molecular genetic basis.. Surv Ophthalmol.

[4] Ahuja Y, Kohl S, Traboulsi EI (2008). CNGA3 mutations in two United Arab Emirates families with achromatopsia.. Mol Vis.

[5] Kaupp UB, Seifert R (2002). Cyclic nucleotide-gated ion channels.. Physiol Rev.

[6] Kohl S, Marx T, Giddings I, Jagle H, Jacobson SG, Apfelstedt-Sylla E (1998). Total colourblindness is caused by mutations in the gene encoding the alpha-subunit of the cone photoreceptor cGMP-gated cation channel.. Nat Genet.

[7] Kohl S, Baumann B, Broghammer M, Jagle H, Sieving P (2000). Mutations in the CNGB3 gene encoding the beta-subunit of the cone photoreceptor cGMP-gated channel are responsible for achromatopsia (ACHM3) linked to chromosome 8q21.. Hum Mol Genet.

[8] Milunsky A, Huang XL, Milunsky J, DeStefano A, Baldwin CT (1999). A locus for autosomal recessive achromatopsia on human chromosome 8q.. Clin Genet.

[9] Wissinger B, Gamer D, Jagle H, Giorda R, Marx T (2001). CNGA3 mutations in hereditary cone photoreceptor disorders.. Am J Hum Genet.

[10] Aligianis IA, Forshew T, Johnson S, Michaelides M, Johnson CA (2002). Mapping of a novel locus for achromatopsia (ACHM4) to 1p and identification of a germline mutation in the alpha subunit of cone transducin (GNAT2).. J Med Genet.

[11] Kohl S, Baumann B, Rosenberg T, Kellner U, Lorenz B (2002). Mutations in the cone photoreceptor G-protein alpha-subunit gene GNAT2 in patients with achromatopsia.. Am J Hum Genet.

[12] Thiadens AA, den Hollander AI, Roosing S, Nabuurs SB, Zekveld-Vroon RC (2009). Homozygosity mapping reveals PDE6C mutations in patients with early-onset cone photoreceptor disorders.. Am J Hum Genet.

[13] Chang B, Grau T, Dangel S, Hurd R, Jurklies B (2009). A homologous genetic basis of the murine cpfl1 mutant and human achromatopsia linked to mutations in the PDE6C gene.. Proc Natl Acad Sci U S A.

[14] Burns ME, Arshavsky VY (2005). Beyond counting photons: trials and trends in vertebrate visual transduction.. Neuron.

[15] Matveev AV, Quiambao AB, Browning FJ, Ding XQ (2008). Native cone photoreceptor cyclic nucleotide-gated channel is a heterotetrameric complex comprising both CNGA3 and CNGB3: a study using the cone-dominant retina of Nrl-/- mice.. J Neurochem.

[16] Peng C, Rich ED, Varnum MD (2004). Subunit configuration of heteromeric cone cyclic nucleotide-gated channels.. Neuron.

[17] Johnson S, Michaelides M, Aligianis IA, Ainsworth JR, Mollon JD (2004). Achromatopsia caused by novel mutations in both CNGA3 and CNGB3.. J Med Genet.

[18] Pang JJ, Alexander J, Lei B, Deng W, Zhang K (2010). Achromatopsia as a potential candidate for gene therapy.. Adv Exp Med Biol.

[19] Michalakis S, Muhlfriedel R, Tanimoto N, Krishnamoorthy V, Koch S (2010). Restoration of Cone Vision in the CNGA3(-/-) Mouse Model of Congenital Complete Lack of Cone Photoreceptor Function.. Mol Ther.

[20] Michalakis S, Geiger H, Haverkamp S, Hofmann F, Gerstner A (2005). Impaired opsin targeting and cone photoreceptor migration in the retina of mice lacking the cyclic nucleotide-gated channel CNGA3.. Invest Ophthalmol Vis Sci.

[21] Seeliger MW, Grimm C, Stahlberg F, Friedburg C, Jaissle G (2001). New views on RPE65 deficiency: the rod system is the source of vision in a mouse model of Leber congenital amaurosis.. Nat Genet.

[22] Umino Y, Solessio E, Barlow RB (2008). Speed, spatial, and temporal tuning of rod and cone vision in mouse.. J Neurosci.

[23] Alexander JJ, Umino Y, Everhart D, Chang B, Min SH (2007). Restoration of cone vision in a mouse model of achromatopsia.. Nat Med.

[24] Glushakova LG, Timmers AM, Pang J, Teusner JT, Hauswirth WW (2006). Human blue-opsin promoter preferentially targets reporter gene expression to rat s-cone photoreceptors.. Invest Ophthalmol Vis Sci.

[25] Pang JJ, Chang B, Kumar A, Nusinowitz S, Noorwez SM (2006). Gene therapy restores vision-dependent behavior as well as retinal structure and function in a mouse model of RPE65 Leber congenital amaurosis.. Mol Ther.

[26] Boye SE, Boye SL, Pang J, Ryals R, Everhart D (2010). Functional and behavioral restoration of vision by gene therapy in the guanylate cyclase-1 (GC1) knockout mouse.. PLoS One.

[27] Pang JJ, Boye SL, Kumar A, Dinculescu A, Deng W (2008). AAV-mediated gene therapy for retinal degeneration in the rd10 mouse containing a recessive PDEbeta mutation.. Invest Ophthalmol Vis Sci.

[28] Pang JJ, Dai X, Boye SE, Barone I, Boye SL (2011). Long-term Retinal Function and Structure Rescue Using Capsid Mutant AAV8 Vector in the rd10 Mouse, a Model of Recessive Retinitis Pigmentosa.. Mol Ther.

[29] Cideciyan AV, Aleman TS, Boye SL, Schwartz SB, Kaushal S (2008). Human gene therapy for RPE65 isomerase deficiency activates the retinoid cycle of vision but with slow rod kinetics.. Proc Natl Acad Sci U S A.

[30] Cideciyan AV, Hauswirth WW, Aleman TS, Kaushal S, Schwartz SB (2009). Vision 1 year after gene therapy for Leber's congenital amaurosis.. N Engl J Med.

[31] Hauswirth WW, Aleman TS, Kaushal S, Cideciyan AV, Schwartz SB (2008). Treatment of leber congenital amaurosis due to RPE65 mutations by ocular subretinal injection of adeno-associated virus gene vector: short-term results of a phase I trial.. Hum Gene Ther.

[32] Jacobson SG, Cideciyan AV, Ratnakaram R, Heon E, Schwartz SB (2011). Gene Therapy for Leber Congenital Amaurosis Caused by RPE65 Mutations: Safety and Efficacy in 15 Children and Adults Followed Up to 3 Years.. Arch Ophthalmol.

[33] Maguire AM, Simonelli F, Pierce EA, Pugh EN, Mingozzi F (2008). Safety and efficacy of gene transfer for Leber's congenital amaurosis.. N Engl J Med.

[34] Kong F, Li W, Li X, Zheng Q, Dai X (2010). Self-complementary AAV5 vector facilitates quicker transgene expression in photoreceptor and retinal pigment epithelial cells of normal mouse.. Exp Eye Res.

[35] Allocca M, Manfredi A, Iodice C, Di VU, Auricchio A (2011). AAV-mediated gene replacement either alone or in combination with physical and pharmacological agents results in partial and transient protection from photoreceptor degeneration associated with beta PDE deficiency.. Invest Ophthalmol Vis Sci.

[36] Petrs-Silva H, Dinculescu A, Li Q, Min SH, Chiodo V (2009). High-efficiency transduction of the mouse retina by tyrosine-mutant AAV serotype vectors.. Mol Ther.

[37] Biel M, Seeliger M, Pfeifer A, Kohler K, Gerstner A (1999). Selective loss of cone function in mice lacking the cyclic nucleotide-gated channel CNG3.. Proc Natl Acad Sci U S A.

[38] Li X, Li W, Dai X, Kong F, Zheng Q (2011). Gene Therapy Rescues Cone Structure and Function in the 3-Month-Old rd12 Mouse: A Model for Midcourse RPE65 Leber Congenital Amaurosis.. Invest Ophthalmol Vis Sci.

[39] Pang J, Boye SE, Lei B, Boye SL, Everhart D (2010). Self-complementary AAV-mediated gene therapy restores cone function and prevents cone degeneration in two models of Rpe65 deficiency.. Gene Ther.

[40] Genead MA, Fishman GA, Rha J, Dubis AM, Bonci DM (2011). Photoreceptor structure and function in patients with congenital achromatopsia.. Invest Ophthalmol Vis Sci.

[41] Pang J, Lei L, Dai X, Shi W, Liu X (2012). AAV-mediated gene therapy in mouse models of recessive retinal degeneration..

[42] Komaromy AM, Alexander JJ, Rowlan JS, Garcia MM, Chiodo VA (2010). Gene therapy rescues cone function in congenital achromatopsia.. Hum Mol Genet.

[43] Yang GS, Schmidt M, Yan Z, Lindbloom JD, Harding TC (2002). Virus-mediated transduction of murine retina with adeno-associated virus: effects of viral capsid and genome size.. J Virol.

[44] Jacobson SG, Acland GM, Aguirre GD, Aleman TS, Schwartz SB (2006). Safety of recombinant adeno-associated virus type 2-RPE65 vector delivered by ocular subretinal injection.. Mol Ther.

[45] Zolotukhin S, Potter M, Zolotukhin I, Sakai Y, Loiler S (2002). Production and purification of serotype 1, 2, and 5 recombinant adeno-associated viral vectors.. Methods.

[46] Deng WT, Sakurai K, Liu J, Dinculescu A, Li J (2009). Functional interchangeability of rod and cone transducin alpha-subunits.. Proc Natl Acad Sci U S A.

