# Correction: AAV-Mediated Cone Rescue in a Naturally Occurring Mouse Model of CNGA3-Achromatopsia

**DOI:** 10.1371/annotation/29fb0ebc-b1c7-4d05-bd70-d29b10299df4

**Published:** 2014-01-06

**Authors:** Ji-jing Pang, Wen-Tao Deng, Xufeng Dai, Bo Lei, Drew Everhart, Yumiko Umino, Jie Li, Keqing Zhang, Song Mao, Sanford L. Boye, Li Liu, Vince A. Chiodo, Xuan Liu, Wei Shi, Ye Tao, Bo Chang, William W. Hauswirth

A grant from the NIH that funded the last author's work for the article is incorrect. The grant number is: R01-EY19304. 

